# Principles of open source bioinstrumentation applied to the poseidon syringe pump system

**DOI:** 10.1038/s41598-019-48815-9

**Published:** 2019-08-27

**Authors:** A. Sina Booeshaghi, Eduardo da Veiga Beltrame, Dylan Bannon, Jase Gehring, Lior Pachter

**Affiliations:** 1 Department of Mechanical Engineering, California Institute of Technology, Pasadena, CA 91125 USA; 2 Department of Biology & Biological Engineering, California Institute of Technology, Pasadena, CA 91125 USA; 3 Department of Computing & Mathematical Sciences, California Institute of Technology, Pasadena, CA 91125 USA

**Keywords:** Lab-on-a-chip, Mechanical engineering

## Abstract

The poseidon syringe pump and microscope system is an open source alternative to commercial systems. It costs less than $400 and can be assembled in under an hour using the instructions and source files available at https://pachterlab.github.io/poseidon. We describe the poseidon system and use it to illustrate design principles that can facilitate the adoption and development of open source bioinstruments. The principles are functionality, robustness, safety, simplicity, modularity, benchmarking, and documentation.

## Introduction

Open source hardware projects^1^ have become increasingly popular in recent years due in part to the rapid evolution of desktop 3D printers and an ecosystem of open source electronic boards like the Arduino and Raspberry Pi systems^2,3^. These developments have spurred growing interest in laboratory instrument open source projects^4–6^ including syringe pumps^7,8^, microscopes^9^, fluorescence imaging devices^10^, micro-dispensers^11^ and single-cell transcriptomics technologies^12^. While cost savings can be an important reason for development of open source hardware^13^, the ability to customize designs for specific applications gives open source projects a unique advantage over commercial solutions. In addition, expanding libraries of designs, software, and commonly used off-the-shelf parts can be shared and adapted across projects, meaning developers are never starting from scratch, even when designing a new instrument. For example, the RepRap project 3D printers borrowed heavily from standard software and hardware Computer Numeric Control (CNC) tools used in machining. As open source designs, electronics boards, software, and parts for 3D printers were continually published and improved, cheap and interchangeable open source hardware and software intended for 3D printing began to be repurposed for new bioinstruments such as liquid handlers^14^, vial handlers and food dispensers^15^, autosamplers^16,17^, and bioprinters^18,19^.

**Figure 1:**
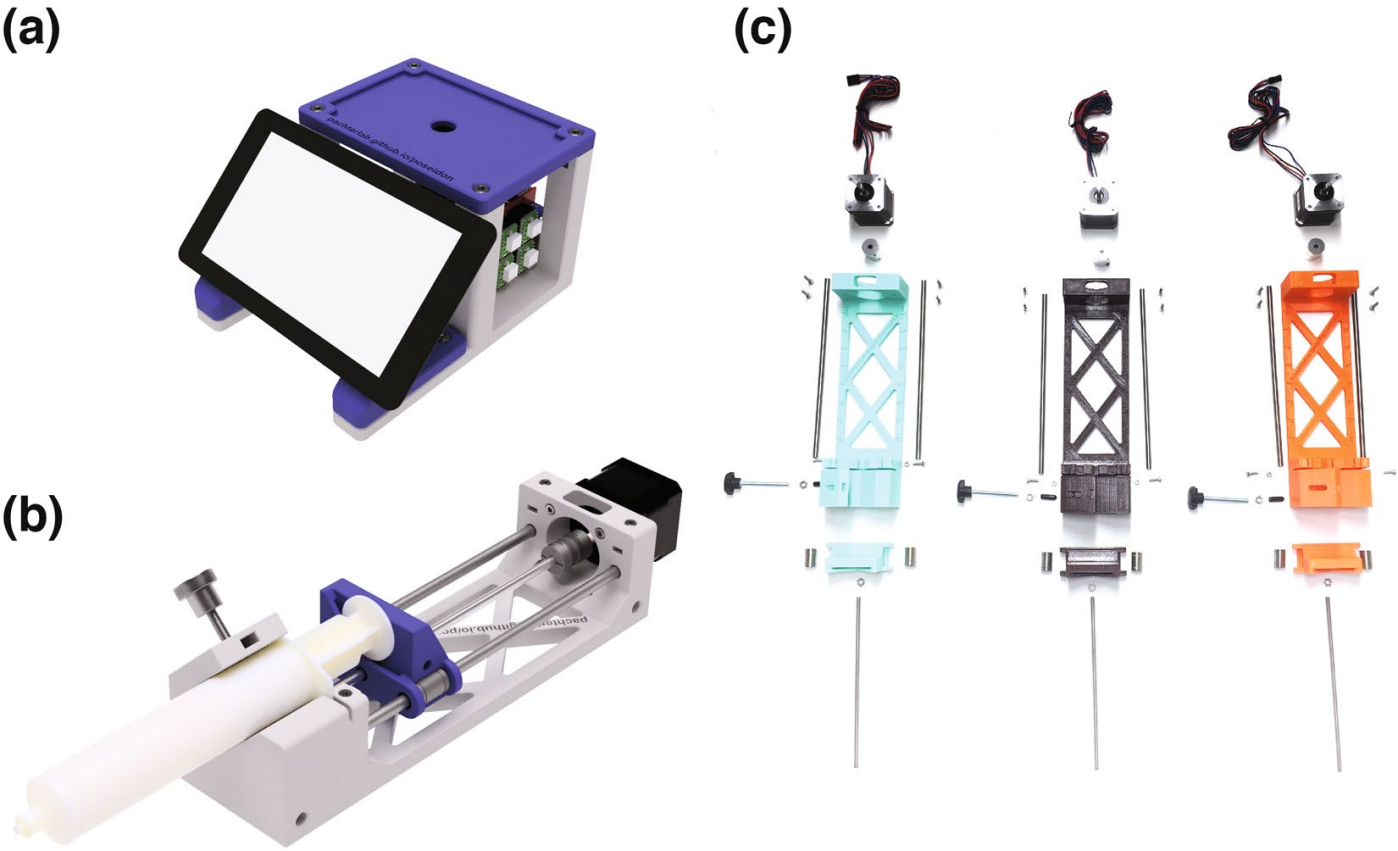
Model of the poseidon system. (**a**) CAD rendering of the microscope station and (**b**) a single syringe pump loaded with a 60 mL syringe. (**c**) Exploded view of all the components needed for assembling three pumps.

Our laboratory has a general interest in developing new methods for high-throughput single-cell applications such as Drop-seq^20^ and inDrops^21^ which rely on precise flow rate control to operate microfluidic devices. The unpredictable landscape of single-cell genomics technology puts a high priority on flexible hardware and software that can be adapted and re-purposed as experiments evolve. The inflexible software interface and functionality offered by commercial systems, and the array of do-it-yourself electronics and instrumentation projects powered by open source hardware, inspired us to develop our own open source multi-syringe pump array and microscope system for low cost microfluidics experiments. The resulting system, which we call poseidon, is based on published open source syringe pumps^7^ and microscope microfluidics stations^12^ but introduces a number of innovations and adapts common 3D printer hardware and software to control the system. Requiring only off-the-shelf components and 3D printed parts, the entire poseidon system including microscope and three syringe pumps can be assembled in less than an hour for less than $400. The poseidon syringe pump array and microscope system is an open source alternative to commercial systems (Fig. 1). The pumps and microscope can be used together for microfluidics experiments, or the pumps can be connected to a computer and used independently. For scientists with tight budgets, the microscope system, which is stand-alone, is an effective solution for basic light microscopy.

**Figure 2:**
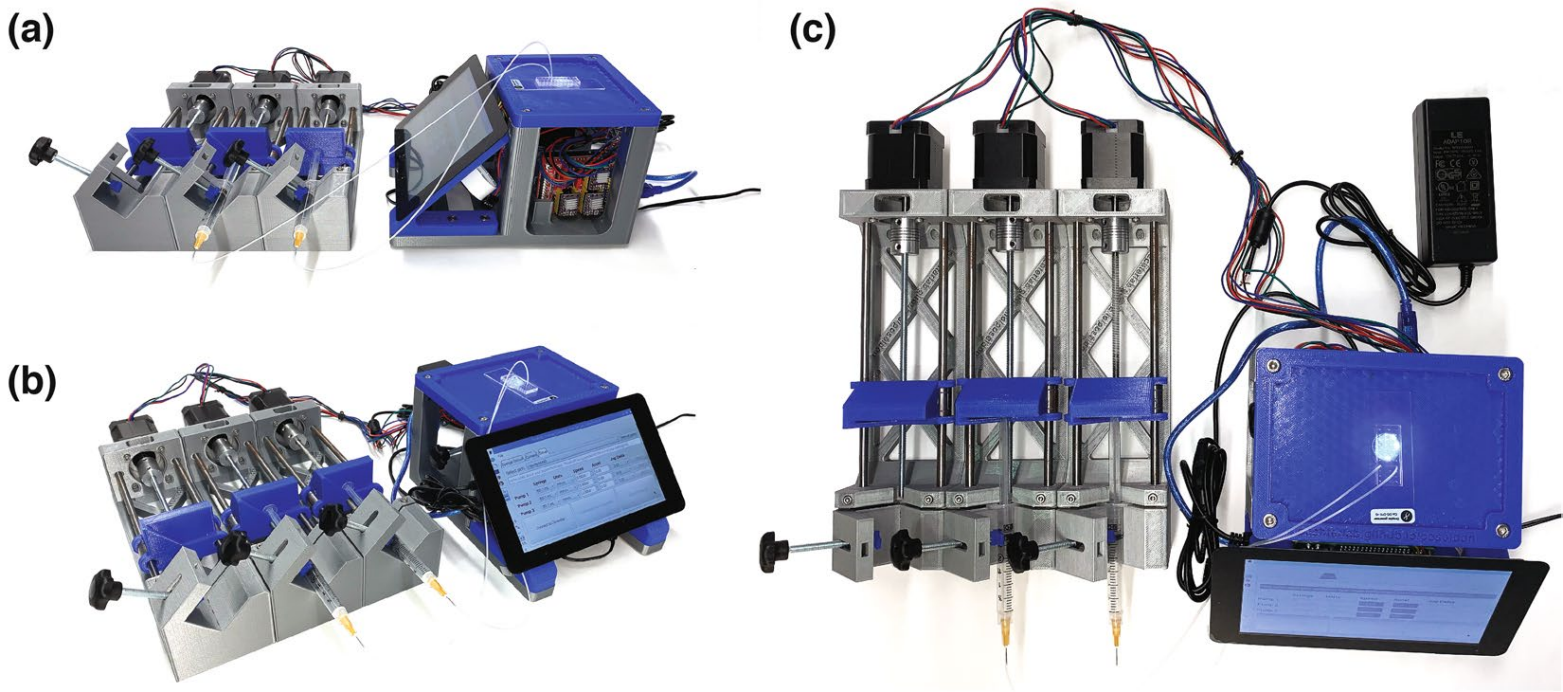
Using the poseidon system. Configuration of the poseidon system for running an emulsion generation microfluidics experiment where only two pumps are used. (**a**) Side view (**b**) angled view (**c**) top view.

**Figure 3:**
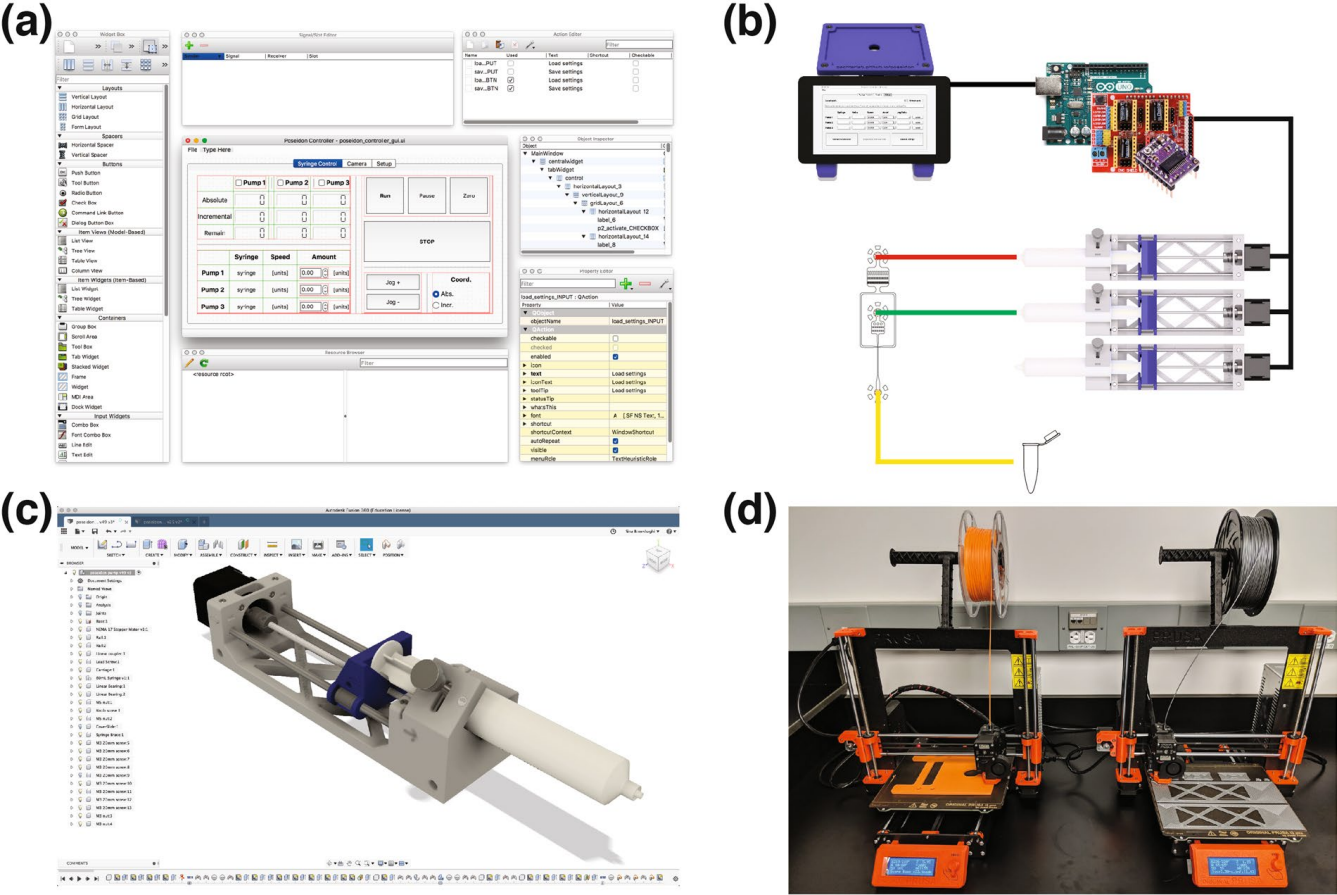
Overview of the tools used for developing the poseidon system. (**a**) The GUI was created using Qt Designer^31^, an open source drag and drop application for organizing buttons that allows users to easily change the GUI interface when adding new functionalities. (**b**) The GUI interfaces with a Python script that controls both the microscope and Arduino via USB. The Arduino controls the stepper motors on each pump using the CNC shield and stepper motor drivers. (**c**) The system’s 3D printed components were designed using Fusion 360^32^, a cloud enabled CAD software that streamlines collaboration and offers free licenses for academics, hobbyists and small businesses. To modify the 3D models users can either work with Fusion 360 or any other CAD software. (**d**) The 3D printed components can be fabricated on any fused filament fabrication (FFF) 3D printer.

The poseidon system uses a Raspberry Pi and touchscreen for the microscope and an Arduino board with a CNC shield to operate up to four pumps simultaneously. Each pump has a stepper motor that drives a lead screw, which in turn moves a sled (mounted on linear bearings) that pushes (infuses) or pulls (aspirates) the syringe plunger. The microscope camera and Arduino use USB connections to connect to the Raspberry Pi or desktop computer (Fig. 2). The system was developed using readily available tools: Autodesk Fusion 360 for CAD, Python 3 and PyQT for software, 3D printers for fabricating custom hardware pieces, and off the shelf electronics and hardware parts (Fig. 3).

The poseidon system repository is available under a BSD 2-clause license at https://github.com/pachterlab/poseidon. For reproducibility and ease of adoption we included direct links to the specific parts used for poseidon, in the GitHub repository. The following components are available:

1. 3D models and Computer Aided Design (CAD) files of the 3D printed components.
2. Pump controller and Graphical User Interface (GUI) software to control the Arduino.
3. Arduino firmware to relay commands via USB to drive the motors.

As we invested more time into poseidon, we realized that the impact of many open source bioinstruments is limited by unintentionally restrictive design decisions and inadequate documentation that discourages adoption by others. This is perhaps unsurprising as most projects are conceived and realized by non-expert developers who are themselves end users. It is with this community in mind that we present a set of guiding design principles specifically tailored to open source bioinstruments. The principles are a synthesis of our experiences designing the poseidon system from the ground up with ease of adoption as our goal. It is our intention that future developers can apply these principles from inception through testing to produce more robust, flexible systems that are more likely to be adopted, modified, and improved by the broader community. Here we detail these design principles using the poseidon system as an illustrated example.

## Design Principles

We strove to produce a bioinstrument that could be readily implemented and modified by others: users and designers who could improve and expand on the system. We considered that bioinstrument users generally fall into two categories: i) those who want to adopt a design and use it in a straightforward manner, and ii) those who want to tweak, improve, and adapt designs to their needs, utilizing the instrument for new use cases. While cost is one motivation for developing and using open source instruments, low cost alone cannot drive the adoption of a project for these two groups. A successful open source instrument appeals to the needs of basic and advanced users by adhering to a set of clear design principles: functionality, robustness, safety, simplicity, modularity, benchmarking, and documentation. Adhering to these principles from the beginning of the design-build-test cycle will result in improved bioinstruments ready for further development and use by others.

### Functionality: developing for an application

In engineering, a functional requirement defines a specific metric that a hardware or software system must achieve. The idea of being “good enough” attempts to capture the many design decisions that can be made during the design-build-test cycle as developers consider the tradeoffs between utility, precision, accuracy, speed, cost, and complexity that are acceptable given the application. The poseidon system needed to achieve the following functional requirements for use in microfluidic applications:

1. The syringe pumps needed to be precise enough to make monodisperse emulsions on droplet generation microfluidics chips, with flow rates on the order of 1 mL/hr.
2. The microscope needed to have sufficient magnification to examine the emulsions and view the microfluidic device during operation.
3. The hardware and control software needed to be able to run at least three pumps independently.
4. The software interface needed to be simple and allow users to easily change flow rates, select syringe type or diameter, and perform gradient pumping.
5. The software needed to operate the microscope.

These were the minimum requirements that were specified before we began developing poseidon. A similar list of specific requirements is a necessary starting point for any bioinstrumentation project. After designing hardware that should be able to meet these objectives, we ensured the pumps operated reliably with flow rates ranging from a few hundred microliters per hour up to several hundred milliliters per minute and we selected an inexpensive USB microscope that reliably imaged our microfluidic device. The 0.3 MP microscope uses a CMOS sensor and has eight dimmable LEDs; the microscope can be readily swapped with any USB compatible microscope. Representative images are in S2.

### Robustness: designing with variation in mind

Robustness encompasses not only mitigating the possibility of failure during operation but also ensuring a construction process that tolerates variability in the components. This is particularly important in biology applications where instruments must frequently work in varying physical conditions and with variable input. Ensuring robustness took considerable time, demanding attention to small details and repeated testing. For example, much open source hardware relies on 3D printed components that can introduce variability when printed on different printers. Mechanical tolerance was built into the 3D printed parts over the course of many design-build-test cycles, for example by modifying the print settings to allow for a press fit of the syringe into the pump. During testing, we discovered an unforeseen hardware issue: when there was too much sliding resistance on the carriage, the linear rods displaced and the printed plastic body bent. To stop the bending, we designed a reinforced body and secured the linear rods with set screws. This level of refinement is to be expected for any bioinstrument, and potential developers should be prepared for several design cycles to create an adoptable device.

On the software side, robustness demands testing to minimize user operation error and to ensure correct functionality. The software must be installed and tested on multiple operating systems to verify operation is as expected. In parallel, internet-capable devices such as the Raspberry Pi should be appropriately set up to to avoid internet-based attacks. Once the poseidon pumps were being used for experiments in our lab and others, usability issues became apparent. For example, one version of the software configured the stepper motors to use a different microstepping than the hardware had configured, an error which the users encountered during their experiments by observing incorrect flow rates. Using the software during an experiment also revealed small usability issues that had to be corrected, such as using a drop-down menu for choosing the flow direction instead of using a “+” or “−” sign in the displacement amount box. These improvements are relatively minor on their own, but we believe the sum total of such small modifications has an outsized impact on potential adopters testing out an unfamiliar system for the first time.

### Safety: communicating hazards to users

Safety critically important for robust device operation and must be carefully considered in a laboratory context. When designing an open source bioinstrument one should always be aware of the health, fire, chemical, and biological hazards present in the laboratory, and other hazards that could arise during instrument construction, normal operation, and possible malfunction. The US Occupational Safety and Health Administration (OSHA) provides guidelines on hazards present in the laboratory environment^22^ and those arising from mechanical equipment operation^23^. Additionally, the International Organization for Standardization (ISO) has developed comprehensive standards on machinery safety^24^. Material Safety and Data Sheets should be used in tandem with these guidelines to design instruments that are robust to hazardous conditions, keeping the user safe.

Certain hazards can arise during the operation of the poseidon syringe pump system that are similar to those encountered when operating other equivalent devices. These include electrical shocks, clogged lines creating pressurized liquids, and material compatibility.

The poseidon syringe pump system uses 3D printed PLA plastic and standard off the shelf components which do not pose a health hazard if handled correctly. Improper handling of plastics however can pose major safety concerns. Designers should consider how their instrument operates when used under elevated temperatures exceeding the melting point of the plastic or under high stress exceeding the yield strength of the plastic. Initial tests of the poseidon syringe pump showed excessive bending of the syringe pump body which we mitigated by reinforcing the body with set screws and a thicker base. We also considered forces induced on the syringe pump due to clogging. To mitigate the possibility of catastrophic failure we set the reference voltage on the motor controller such that the motor would stall when the pressure in a 1 mL syringe reaches 4 MPa, prior to any failure occurring^25^, and well below the ultimate tensile strength of the PLA plastic used in the current design^26^.

The chemical properties of the materials used in designing instrument parts should be considered^27^ if one is designing an instrument that could come in contact with organic solvents. We note that the PLA plastic used is compatible with most solvents^28^. One benefit of open source 3D printable designs is that there are a number of 3D printing materials that are chemically compatible with many types of standard wet lab environmental conditions and hazards^28,29^. Finally, in the development of any open source bioinstrument, after identifying safety requirements it is important that hazards and safe operating procedures be clearly communicated. We describe the safety aspects of the poseidon system on the project Github page.

### Simplicity: making it easy to source, build, and operate

Simplicity and ease-of-use are essential for the adoption of bioinstruments. Sourcing components for a design should be as easy as possible, prioritizing off-the-shelf components during development and incorporating harder to find parts only if necessary for the application at hand. An accurate and up-to-date bill of materials (BOM), with ideally more than one vendor for each part, simplifies purchasing and leads to easier adoption. For the poseidon system, we ensured that users would be able to purchase all the components from Amazon. During assembly, it is important to recognize that using specialized equipment - even soldering a circuit board - may be a barrier to adoption. While such specialized assembly processes are sometimes unavoidable, simplicity is paramount. An excellent way to assess the difficulty of assembly is to have people unfamiliar with the project perform the assembly using only the documentation available. With the poseidon system it was possible to design around most of these constraints, and we verified that assembly of a single pump by a new user following the instruction video takes less than 15 minutes, requiring only pliers and screwdrivers.

Simplicity considerations also apply to software. For example, minimizing dependency on external software libraries simplifies installation and avoids versioning issues. From a user’s perspective, having a single executable file for the software is ideal. We compiled the Python scripts into single-click executable files for Mac, Windows, and Linux. After testing we realized that a flexible user interface design was critical to develop software that minimized user error. The original rigid custom GUI code did not allow us to easily resize buttons, change button layout, or add new functionalities. Using Qt Designer, a drag and drop GUI creator, we could overcome these challenges and create a basic, functional user interface that is touch-screen and click-button compatible. Additionally, the GUI can easily be adapted and modified for the needs of future adopters. The custom poseidon Arduino firmware needs be loaded onto the Arduino Uno board following simple instructions. If users wishes to use a Raspberry Pi to operate poseidon, installation requires flashing an SD card with the official version of the Raspbian OS image.

### Modularity: building independent and individual units

Because some users will want to adapt a design to new use cases, it is important to consider how easily a design can be taken apart, tweaked, and re-purposed. A modular design with independent components that can be interfaced with each other is easier to re-purpose and improve on than a tightly integrated device. When a design is not modular, developing new features may require a complete redesign. This is the problem that led us to develop poseidon in the first place - our commercial, highly integrated system was too rigid to meet our changing needs. The software could not be improved or modified, and the small integrated touchscreen interface, marketed as an advantage^30^, was a hindrance to routine operation. In the case of poseidon, some users might want to add additional features to the pumps such as an electronic end stop. The modularity of the Arduino board makes this change straightforward and simple to implement.

A design is also easier to modify when common and standardized parts and connectors are used. Standardization is ubiquitous in both open source hardware and software projects. Open source 3D printers use a common set of screws, rods, extruding nozzles, and electronics. Often these printers are variations of a few common, popular, and proven designs. The standardization of 3D printer parts means that they, in turn, can be readily adapted for new use cases. Almost all of the components used in the poseidon system can be found in open source desktop 3D printers.

### Benchmarking: validating with standard protocols

Users need to know the degree to which an instrument design is applicable to the problem at hand. Thus it is important to describe protocols where instruments have been applied and provide benchmarking results. Open source instruments may not always perform as well commercial systems, but may still be good enough for many applications. Direct comparison with commercial instruments and clear identification of device shortcomings, or features still in development, is important to instill confidence in the device.

**Figure 4:**
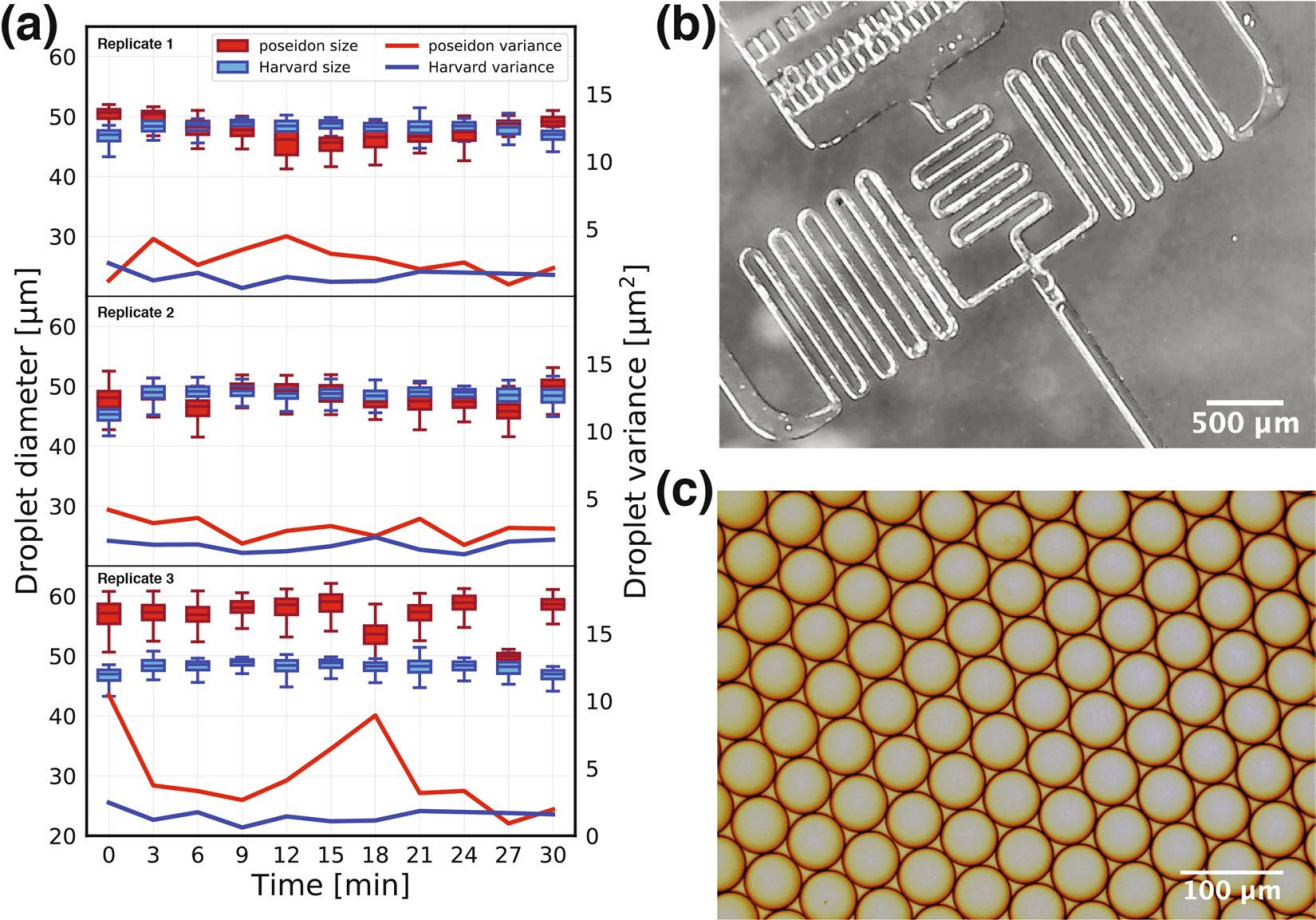
Benchmarking the poseidon system against the Harvard Apparatus system. Using a droplet generation chip we compared the droplet diameters between two systems. (**a**) A droplet size of 58 *μ*m in diameter is expected for the given flow rates. The variance in the sizes of the droplets created with the two systems is comparable. (**b**) A microfluidic droplet generation chip imaged using the poseidon microscope. (**c**) Example of a monodisperse emulsion produced by the poseidon system and imaged with a Motic AE31 Trinocular Inverted microscope.

The poseidon system has been successfully used in generating monodisperse emulsions using the inDrops droplet generation device (Fig. 4). We achieved targeted droplet size with small variance in droplet diameter. Importantly, we directly compared poseidon with the commercial array from Harvard Apparatus (catalog numbers 2401-408 and 70-3406), demonstrating comparable variance in droplet diameter. The poseidon system can be operated at a range of flow rates (Table S1). By physically adding or removing the microstepping jumpers on the CNC shield board users can access an even wider range of flow rates, so that the same system can be used for high-precision microfluidics experiments and high flow rate applications such as protein purification.

### Documentation: describing the design completely

Clear instructions and documentation are essential to facilitate rapid and painless assembly. Videos, photographs and written descriptions are fundamental for showcasing a design and ensuring adoption. For assemblies, videos are often the most helpful documentation for users and do not take much time and effort to produce. Videos also clearly convey how much effort and time users should expect to invest in assembly. Documentation enables faster and easier understanding of design.

Users who want to modify a design will additionally benefit from understanding design decisions - both those motivated by technical considerations and those motivated by user feedback. How to implement each feature is the result of thought and iteration from the designer, but what is learned may not be readily apparent in the final designs. Documentation of lessons learned, and insight into why design features were implemented a certain way is important; sometimes modifications that seem to be an improvement will create a failure mode that is not readily apparent.

For the poseidon system, multiple build videos are available on YouTube showing the entire assembly process. In making the poseidon documentation website, we also strove to use clearly labeled photos of the hardware with short written instructions, as this makes it easier for prospective users to grasp the design and expected time investment at a glance. The development and documentation of open source projects benefits tremendously from making use of version control repositories, which streamlines remote collaboration and development tracking. For the poseidon system, we used the online repository GitHub, which allows for version control and documentation of each change made and makes it simple to create user guides and device documentation as can be seen at https://github.com/pachterlab/poseidon.

## Conclusion

**Figure 5:**
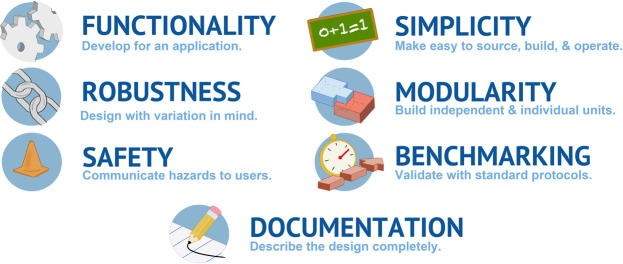
Summary of the design principles for open source bioinstrumentation.

Success and adoption of open source software demonstrates that reliable and powerful technologies can be realized through community development and improvement. We have developed a modular, highly customizable syringe pump array and USB microscope system, poseidon, with potential for broad application across the biological and chemical sciences. Syringe pumps can be used to operate microfluidic chips, control the chemical environment of a bioreactor, purify proteins, precisely add reagents to chemical reactions over time, or dispense specific amounts of fluid for any number of applications that require precise control of fluid flow. We have benchmarked the system against a commercial alternative in a demanding application: microfluidic emulsion generation. In developing the poseidon system as an open source hardware device we have illustrated seven design principles that we hope can facilitate successful development of open source hardware devices (Fig. 5). Adhering to these principles from the outset of a project will maximize the chance of community adoption and spur further improvements.

## Supplementary information

Supplementary PDF

## Data Availability

Testing data is available at https://github.com/pachterlab/poseidon.
