# Author Correction: Principles of open source bioinstrumentation applied to the poseidon syringe pump system

**DOI:** 10.1038/s41598-023-42035-y

**Published:** 2023-09-08

**Authors:** A. Sina Booeshaghi, Eduardo da Veiga Beltrame, Dylan Bannon, Jase Gehring, Lior Pachter

**Affiliations:** 1 Department of Mechanical Engineering, California Institute of Technology, Pasadena, CA 91125 USA; 2 Department of Biology & Biological Engineering, California Institute of Technology, Pasadena, CA 91125 USA; 3 Department of Computing & Mathematical Sciences, California Institute of Technology, Pasadena, CA 91125 USA

Correction to: *Scientific Reports* 10.1038/s41598-019-48815-9, published online 27 August 2019

This Article contains an error in Figure 4, where the replotting of a subset of data in Figure 4a, which pertain to the Harvard dataset is incorrect in panels (1) and (3). The correct Figure 4 and accompanying legend appear below.

**Figure 4:**
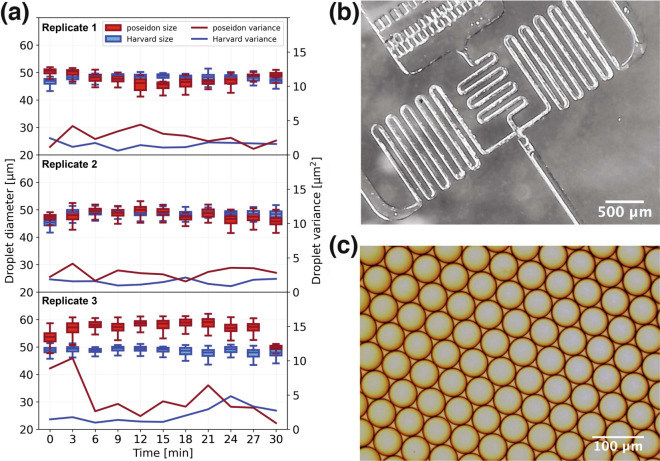
Benchmarking the poseidon system against the Harvard Apparatus system. Using a droplet generation chip we compared the droplet diameters between two systems. (**a**) A droplet size of 58 *μ*m in diameter is expected for the given flow rates. The variance in the sizes of the droplets created with the two systems is comparable. (**b**) A microfluidic droplet generation chip imaged using the poseidon microscope. (**c**) Example of a monodisperse emulsion produced by the poseidon system and imaged with a Motic AE31 Trinocular Inverted microscope.

