# Supplementary material for: Principles of open source bioinstrumentation applied to the poseidon syringe pump system
Source: Sci Rep. 2019 Aug 27;9:12385. doi: 10.1038/s41598-019-48815-9 (PMC6711986; doi:10.1038/s41598-019-48815-9)
Supplement: Supplementary file 1 — Supplementary PDF [file 41598_2019_48815_MOESM1_ESM.pdf]

Supplementary: Principles of open source bioinstrumentation applied to the poseidon syringe pump system

A. Sina Booeshaghi[1], Eduardo da Veiga Beltrame[2], Dylan Bannon[2], Jase Gehring[2,*], and Lior Pachter[2,3,*]

[1]Department of Mechanical Engineering, California Institute of Technology, Pasadena, CA 91125, USA
[2]Department of Biology & Biological Engineering, California Institute of Technology, Pasadena, CA 91125, USA
[3]Department of Computing & Mathematical Sciences, California Institute of Technology, Pasadena, CA 91125, USA
[*]jgehring@caltech.edu, lpachter@caltech.edu

ABSTRACT

The poseidon syringe pump and microscope system is an open source alternative to commercial systems. It costs less than $400 and can be assembled in under an hour using the instructions and source files available at https://pachterlab.github.io/poseidon. We describe the poseidon system and use it to illustrate design principles that can facilitate the adoption and development of open source bioinstruments. The principles are functionality, robustness, safety, simplicity, modularity, benchmarking, and documentation.

Microstep	Syringe Size:	1 mL	3	5	10	20	30	60
1		624	2,084	4,065	5,887	10,261	13,179	19,946
2	Maximum	499	1,667	3,252	4,710	8,209	10,544	15,957
4	Flow Rate	250	834	1,626	2,355	4,104	5,272	7,978
8	[mL/hr]	125	417	813	1,177	2,052	2,636	3,989
16		62	208	406	589	1,026	1,318	1,995
32		31	104	203	294	513	659	997

Table S1. Maximum rates for given syringe and microstepping: Maximum flow rates [mL/hr] for a single pump for a given microstepping and Becton Dickinson (BD) syringe size. At lower flow rates, higher microstepping is desirable for a smoother flow.

Microstep	Steps per Revolution	Precision [μm]	Maximum Speed [mm/s]
1	200	4	10
2	400	2	8
4	800	1	4
8	1600	0.5	2
16	3200	0.25	1
32	6400	0.125	0.5

Table S2. Stepper motor performance: Precision was calculated based on the steps per revolution and the pitch of the lead screw (0.8 mm/revolution). The maximum speed of the carriage was measured and an error of 5.5% ± 1.5% s.d. of the set speed was observed (n = 52).

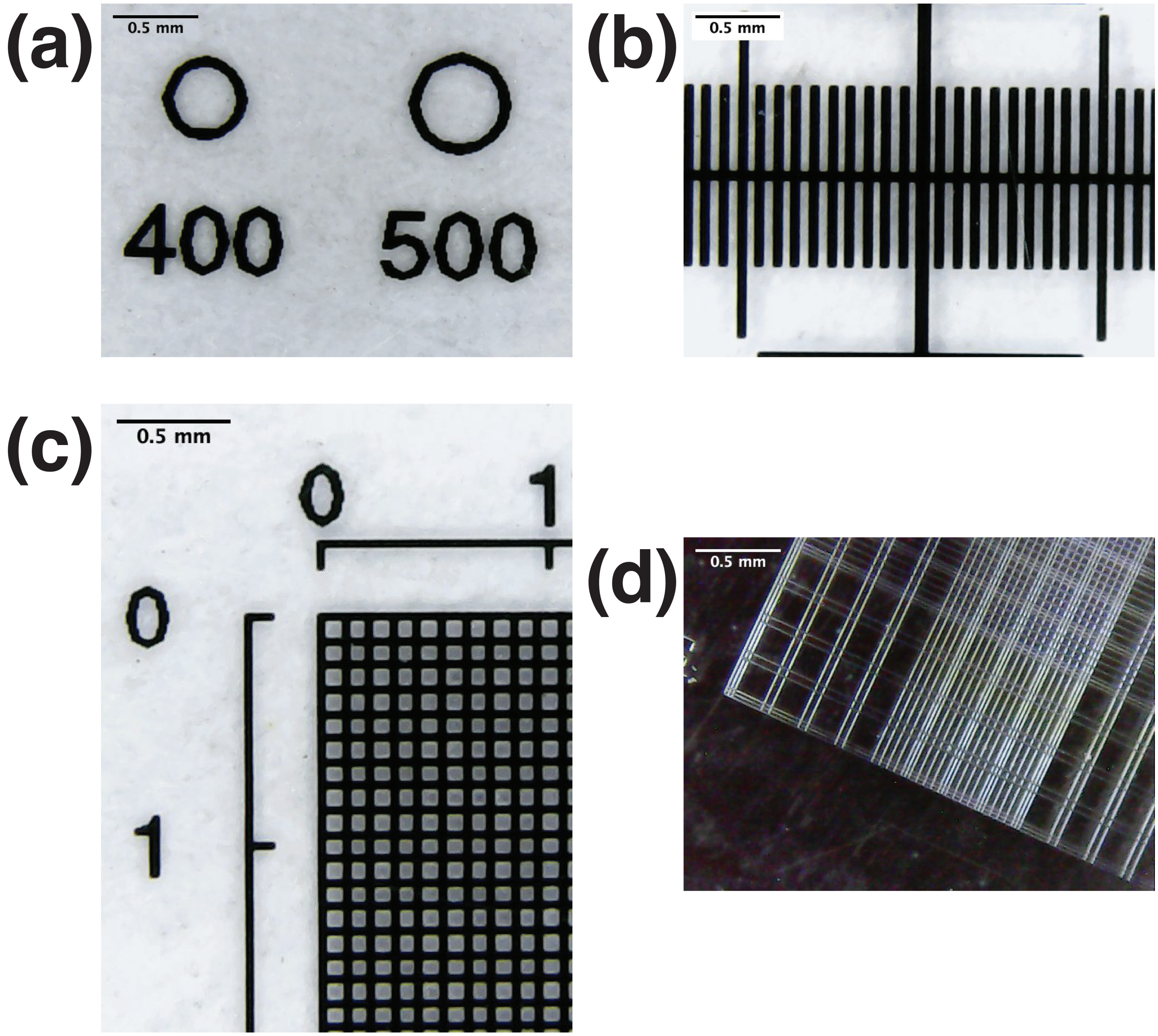

Figure S1. Representative images taken with the poseidon microscope. Images **(a)** - **(c)** are of the calibration slip supplied with the microscope system and image **(d)** is of the Incyto Hemocytomer DHC-B02 (Burker Turk).

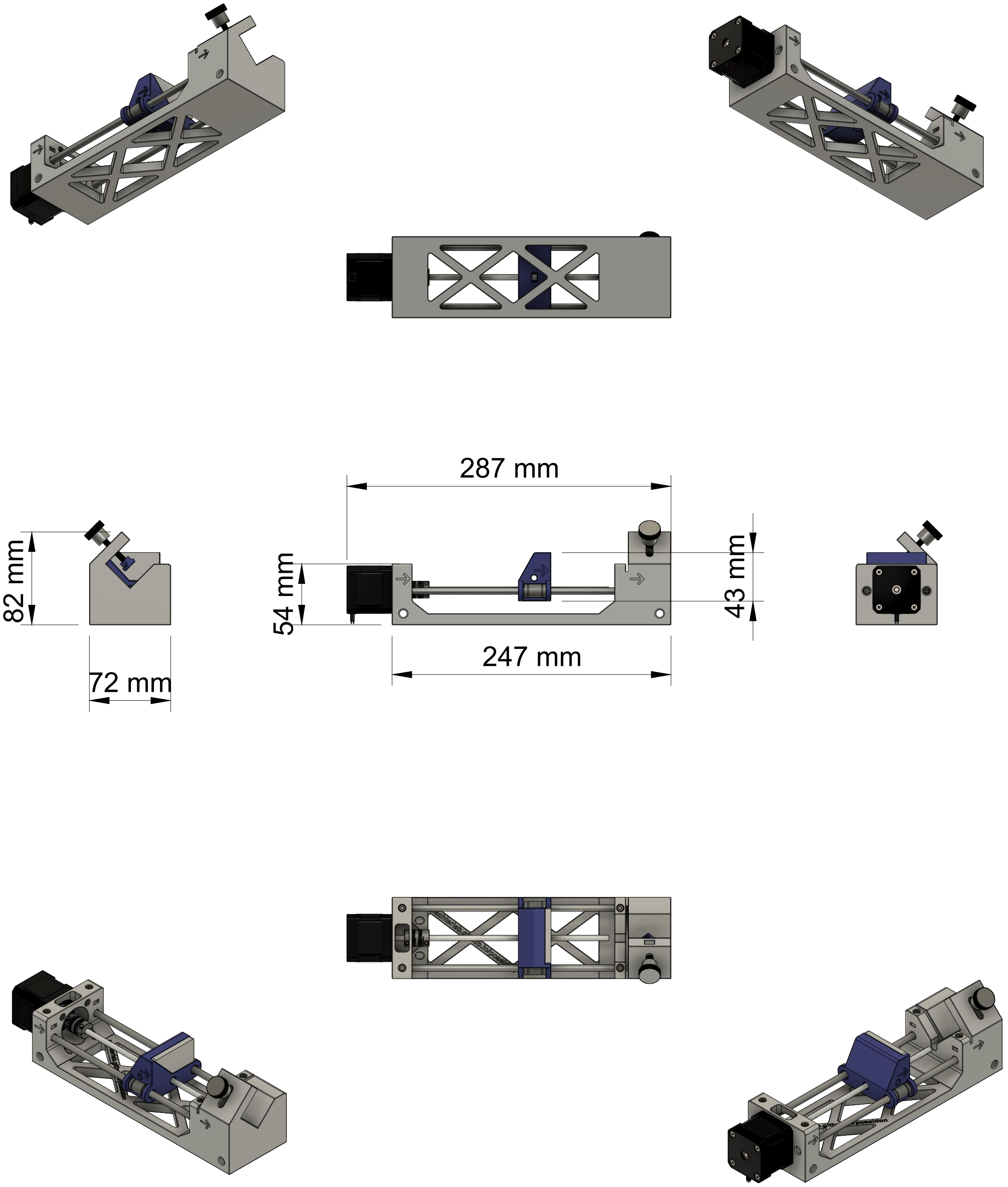

Figure S2. CAD renderings of the poseidon syringe pump. Multiple views and major dimensions are shown.

Parts List		
Item	Qty	Part Name
1	1	3D printed pump body
2	1	NEMA 17 stepper motor
3	2	6mm steel rod, 200mm length
4	1	5mm to 5mm motor shaft coupling
5	1	M5 threaded rod, 170mm length
6	1	3D printed carriage
8	2	6mm linear bearing
9	2	M5 nut
10	1	M5 knob screw
11	1	3D printed cover slide
12	1	3D printed syringe brace
13	8	M3 screw, 20mm length
14	4	M3 nut

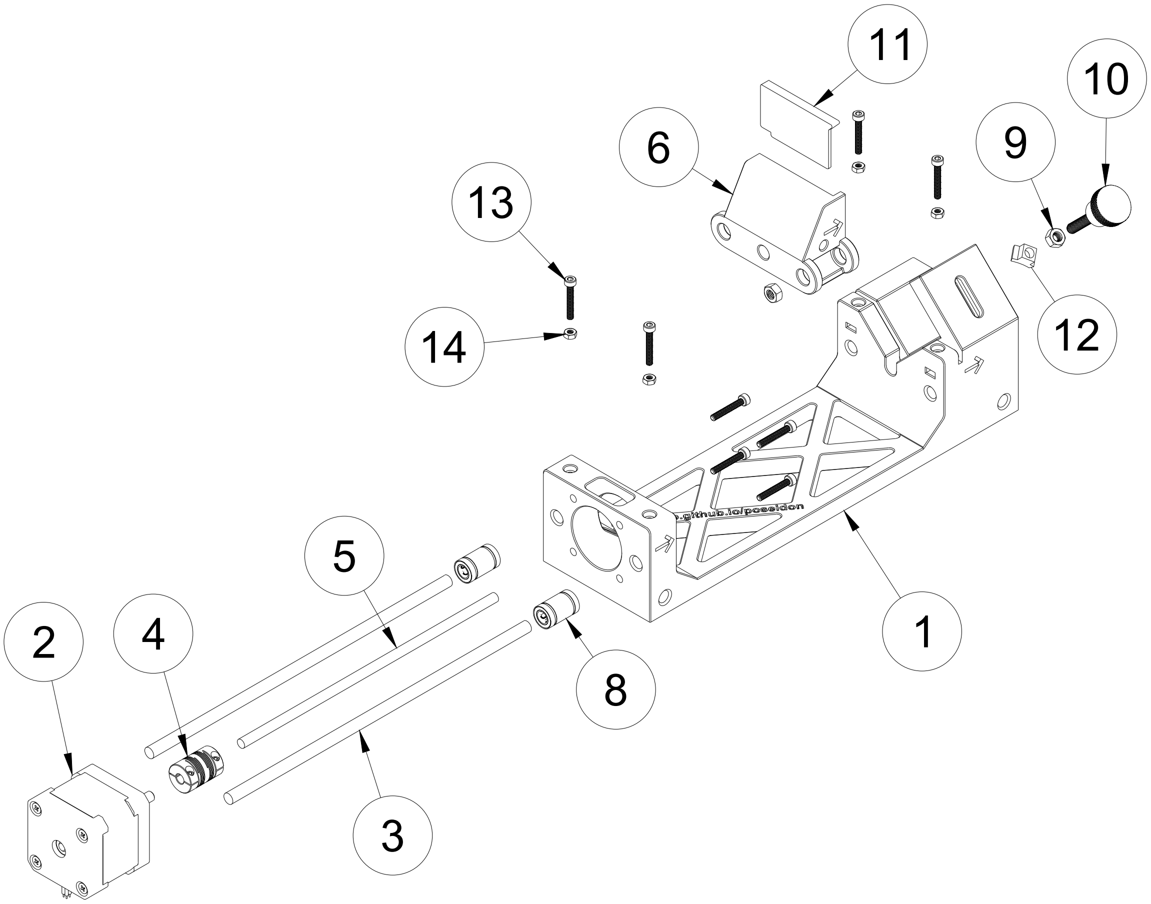

Figure S3. Parts list and exploded view of the poseidon syringe pump.

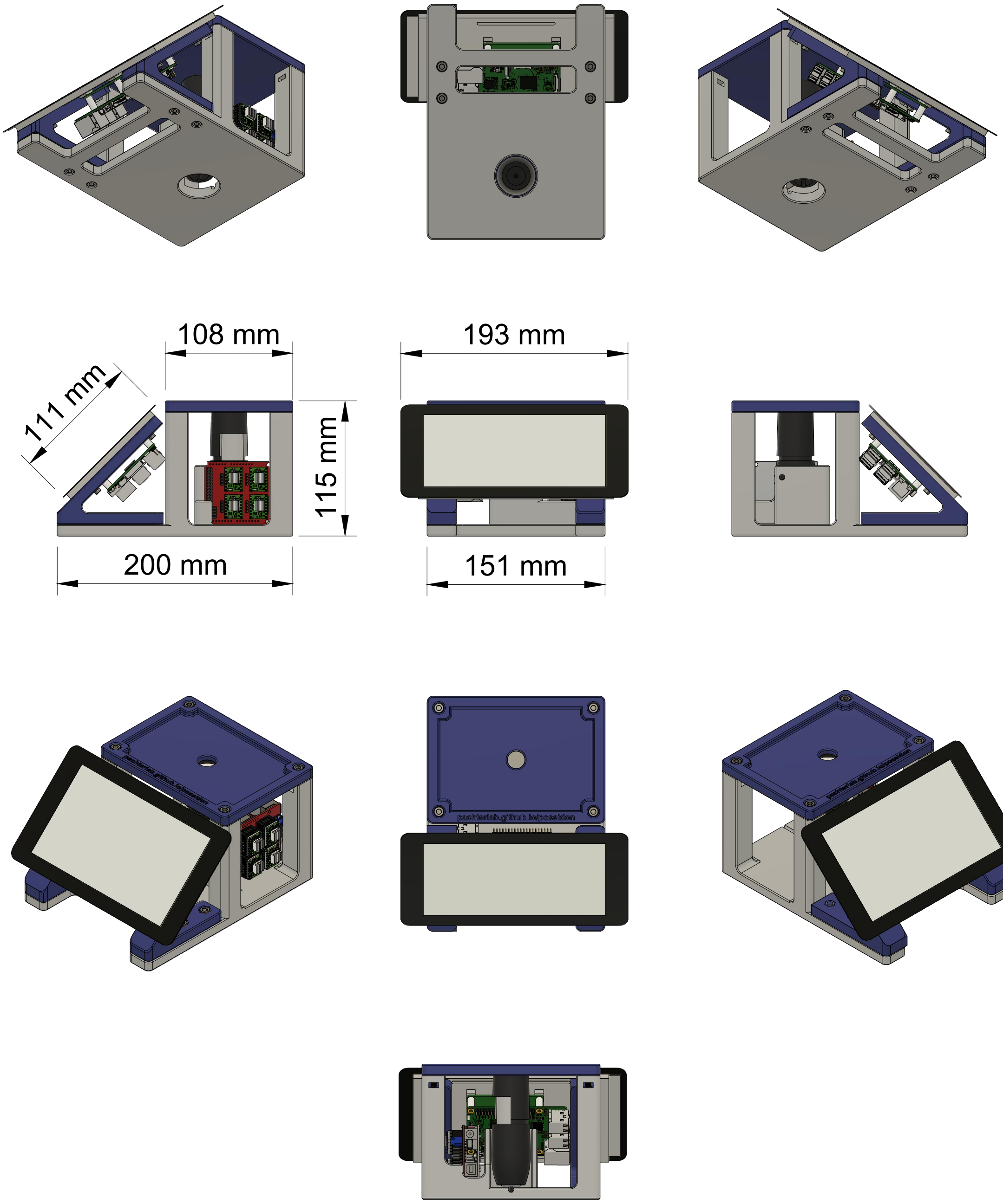

Figure S4. CAD renderings of the poseidon microscope. Multiple views and major dimensions are shown.

Parts List		
Item	Qty	Part Name
1	1	Raspberry Pi board and touchscreen
2	2	3D printed screen leg
3	1	3D printed microscope base
4	1	Arduino UNO
5	1	Arduino CNC shield
6	1	3D printed top stage
7	4	M3 screw, 10mm length
8	8	M5 nut
9	8	M5 screw, 14mm length
10	1	USB microscope
11	1	M5 knob screw

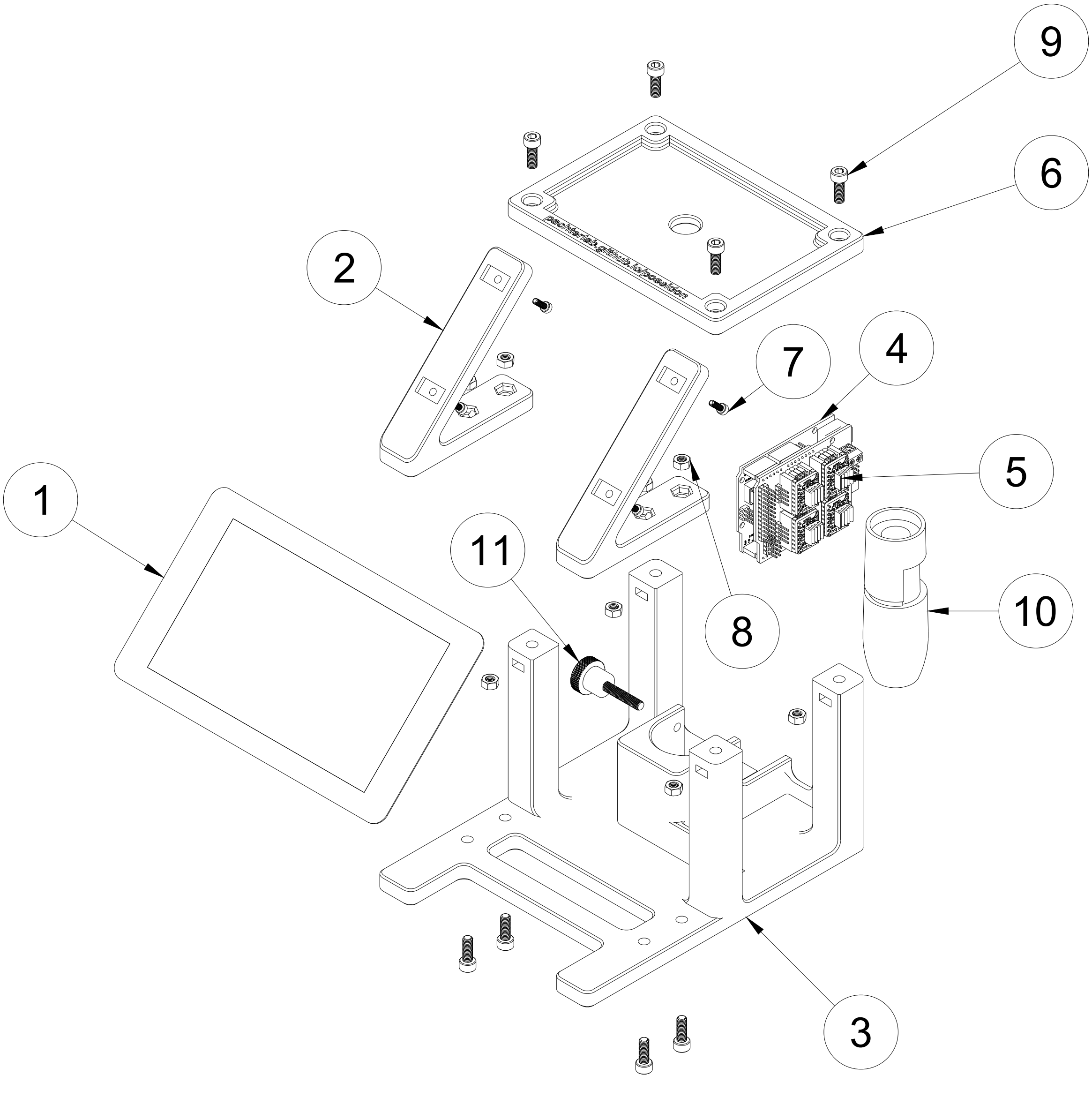

Figure S5. Parts list and exploded view of the microscope.
